# Emotional and Behavioral Problems of Children with ASD—The Lessons That We Learned from the Pandemic

**DOI:** 10.3390/children10060969

**Published:** 2023-05-30

**Authors:** Margarita Stankova, Tsveta Kamenski, Ivan Ivanov, Polina Mihova

**Affiliations:** 1 Department of Health Care and Social Work, New Bulgarian University, 1618 Sofia, Bulgaria; tkamenski@nbu.bg (T.K.); pmihova@nbu.bg (P.M.); 2 College of Veterinary Medicine & Biomedical Sciences, Texas A&M University, College Station, TX 77843, USA; iivanov@cvm.tamu.edu

**Keywords:** autism, COVID-19, special education, emotional and behavioral difficulties

## Abstract

The data available for changes in the behavior and emotional state of children with ASD (autism spectrum disorder) in lockdown situations are controversial and scarce. In our research, we compare results before the first COVID-19 lockdown of 21 children with ASD and 21 typically developing children, four to five years of age with those obtained immediately after. The study attempts to answer the question of whether there are changes in the levels of emotional and behavioral problems in children with ASD after the lockdown and how these new living conditions affect some aspects of their functioning. The instruments used for data analysis are the Childhood Autism Spectrum Test (CAST); Child Behavior Checklist (CBCL); Survey on the consequences of COVID-19 on the life and development of the participants. No significant differences in the emotional and behavioral state of the participants were found, except for attention deficit/hyperactivity problems where ASD children showed lower levels after the lockdown. ASD group parents’ answers to the survey pointed towards more positive consequences of staying at home. Some reported they had more time for learning together, communicating, playing, and assisting the learning process through online therapy. As negatives, the parents of ASD children reported low physical activity, increased time with electronic devices, and time spent with the same people. Caregivers of typically developing children agreed that the lockdown had only negative effects. To conclude, for children with ASD in the study, the lockdown period demonstrated that more time spent with parents in structured everyday activities is an opportunity that can lead to positive results in their behavior.

## 1. Introduction

In 2020, due to the COVID-19 pandemic, schools and kindergartens were closed for students and teaching staff alike in many countries. Because of these measures, both typically developing children and children with special educational needs/SEN transitioned to online education, a model which proved challenging for teachers and specialists involved in the provision for students with ASD. The circumstances that proved to be limiting for students on the spectrum included a significant change in their educational activities and leisure routines and restricted access to medical and psychological support.

The COVID-19 pandemic brought about a considerable change in the lives of families with children on the spectrum, especially when the children were younger or had more profound challenges. The families of children with ASD, especially when the ASD is paired with intellectual disability, had multiple and complex needs. Having to stay at home with partial access to healthcare, while maintaining social distancing, limited parents’, and caretakers’ ability to provide adequate care for their children [1], while specialists struggled to provide therapeutic interventions and consultations remotely [2]. Gaining access to therapy and financing and organizing activities were all found to relate to higher levels of stress in individuals enrolled in more intensive forms of therapy [3]. The parents reported feeling hopeless, lacking support, and that the needs of their children with ASD were heightened by the circumstances [4]. Some of the factors negatively impacting the psychological well-being of autistic children included heightened levels of parental stress and insecurity, illnesses in the family, and low family income [5].

Higher levels of parental distress were observed due to the lockdown [6,7]. Stress and increased anxiety in parents and caregivers exacerbated the emotions and behavior of children, due to the mandatory homestays as well as communicating only with relatives, this influence was expected to be even greater. Additionally, the age of the carers and the lack of other family members who could provide support were found to be quite stressful for the families [8]. Parents of children with ASD faced various challenges during the lockdown, such as working from home or losing their jobs, high workloads, and difficulties keeping up with responsibilities and appointments connected to their children’s conditions without the support of relevant professionals. One area of provision that was particularly affected was the hospital treatment of individuals on the spectrum, with challenges connected to changes in home and leisure routines [9], lower ability to provide professional assistance, and changes in health service delivery [10].

Social distancing and isolation have been consistently connected with negative effects on the psychological well-being of people all over the world. Frustration, loneliness, and uncertainty about the future all contribute to either making anxiety issues in children with ASD more acute or to them developing new ones [11]. The new social rules, necessary in the context of the pandemic, seem to have resulted in changes in behavior and emotional regulation. Some children on the spectrum demonstrated less involvement in play-connected activities and more behavioral challenges during the pandemic [12]. The reality of the pandemic forced children to follow rules that they did not necessarily understand, and they were distrustful of routines such as hand sanitizing, social distancing, and wearing masks outside [13]. Individuals on the spectrum feel calmer in a safe and familiar environment, which is structured and predictable [14]. This, in turn, makes changes and disruptions in the routine stressful. The psychological effects of the COVID-19 pandemic on people with ASD were most likely higher due to the direct influence of the pandemic on social functioning and everyday life. Life in isolation relates to higher anxiety and depression, with people on the spectrum having the additional stress of disruption to their everyday routines. A positive tendency observed in adults with ASD was lower social overload, commonly associated with autism [2]. This could prove to be an important factor when observing emotional and behavioral issues in children. The influence of stress and psychological trauma can be severe in individuals with ASD and intellectual disabilities (ID) due to the difficulty of identifying the symptoms of these [15]. The sleep habits of children with ASD are found to have been disrupted in relation to the pandemic [16].

Despite expectations, some studies show more verbal behaviors in children on the spectrum. This change is perhaps related to the fact that there were more occasions for interactions between family members, especially those involving fathers. Some parents also reported positive changes in every day and self-care skills of their children [17], as well as a positive change in communication and social skills [18]. It is highly likely that this phenomenon is also related to the larger amount of time parents spent teaching their children, combined with a more relaxed approach that relied on the application of methods that support repetition and consolidation of the learning material. Some of the patients on the spectrum who prefer social isolation reported better functioning during the pandemic and lockdown. This is probably more acute for people with social interaction difficulties [19]. However, when considering the benefits of the lockdown, a factor to take into account when planning a therapy intervention is the declining state of psychological functioning at the end of the lockdown [20].

## 2. Services for Children with Developmental Disorders during the COVID-19 Pandemic in Bulgaria

Services provided for children with developmental disorders in Bulgaria include access to various specialists such as speech therapists, psychologists, and special educators [21]. To accommodate the new requirements devised by safety measures during the pandemic, specialists had to adopt new, more interactive, and interesting methods, which could be used in an online environment. These methods had to be adapted for several main purposes: keeping children’s focus on the task at hand, ensuring active inclusion in the learning process leading to measurable results in children’s progress. An additional relevant factor is the specialists’ lack of experience and inadequate training in the application of distance-based therapeutic approaches in an online environment. Despite that, they displayed positive attitudes toward using the advances in technology in their work [22,23,24].

Home learning turned out to be challenging and strange for many children with ASD. Although computers, tablets and similar electronic devices were familiar to them, most had not previously interacted with a specialist or a teacher online and followed their instructions. For children using alternative communication devices and picture exchange, parental support was often essential for them to be able to participate effectively, which, in turn, required more effort and time from the caregivers [2]. A UNICEF study shows that only 35% of kindergartens in Bulgaria continued to actively interact with parents, and only 63% of the inclusion officers continued their regular work with children with special needs during the lockdown, which represents half or even less of the children usually in their care [25]. The pandemic also raised the issues of communication and collaboration between families and specialists who work towards improving the lives of people on the spectrum, especially in times when their specific needs seemed to be heightened by circumstances [26]. Change was challenging for specialists as well, as they had to adapt their programs and methods to accommodate children with ASD and their communication needs. The result seemed to be limited access to services such as speech therapy, physical therapy, and occupational therapy [1], which are particularly important for children under the age of five [27].

The challenges of the pandemic clearly showed the need for more creative adjustments by support teams in order to adapt to the new requirements of an environment in which they had to provide services to children with ASD, particularly oriented towards overcoming the changes in familiar routines [28]. Some of the benefits of online education are improvements in students’ computer skills, motivating teachers to seek out more interactive teaching methods, opportunities to engage with and pay individual attention to each student, easier access to education from any place in the country, more time for sleep, and less travel time. Some of the positive aspects of online learning for ASD children of preschool age were the improvement of their skills in adapting to online interventions, the continuous interaction with their parents and the online support during the lockdown [29]. The application of online interventions and phone consultations also proved effective [30]. Despite these benefits, many studies of the effects of online education conclude that it had some negative tendencies: no personal contact with the teacher, lack of social communication and group interaction at school, distractibility at home and more time to study, more homework. In addition, they reported challenges related to the technical competencies of some teachers and specialists, more time for teachers to prepare and plan their lessons and difficulties providing computers and media to access classes as well as the need for a good Internet connection. There is an additional difficulty when looking at the experiences of children with SEN, as well as those with ASD, related to online education, namely the reliance of the teaching methods used on the parents’ ability to become involved in the learning process. Other issues regarding children with ASD are the caregivers’ skill in working with devices, the difficulties in attention regulation (which are difficult to overcome in front of the computer), the lower chances for interaction with other typically developing children (which is detrimental to their social skills), and difficulties maintaining learning motivation [31].

Studies suggest that many, but not all, ASD children displayed an increase in behavioral disruptions during the first outbreak of COVID-19 [32], potential reasons caused by online training and virtual communication with therapists, others to staying at home, others to increased anxiety and stress in parents [33]. The goal of the current study is to provide information about the levels of identified behavioral and emotional disturbances in children with ASD during the first lockdown. Our aim is also to identify some other challenges and consequences from the lockdown for children and parents and draw conclusions about future strategies and interventions that could be used during and out of pandemic circumstances. As the information on changes in behavioral and emotional difficulties of children with ASD during the pandemic is often controversial, the present comparison attempts to shed light on both impaired and potentially improved elements of behavior and emotional regulation, and to discuss the reasons for these differences. The lockdown was a unique experience for which no one was prepared, online learning happened ad hoc for children, professionals, and parents. These new circumstances are expected to affect different parameters of behavior and emotion regulation, and from these differences some important conclusions can be drawn about our overall attitude and therapeutic approach towards children with ASD and their families.

## 3. Material and Methods

### 3.1. Study Design

To check the variance in behavioral and emotional difficulties in children with ASD during the first lockdown, we compared the results from a study carried out before the lockdown with ASD children (N = 21) and typically developing children (N = 21).

Despite the small sample, our study is unique as, coincidentally and in connection to another planned piece of research, we tested both groups in February 2020, immediately before the outbreak of COVID-19. At the same time, we had the opportunity to examine the same group of children with ASD after the first three-month lockdown, when all schools, children’s centres, kindergartens, and many other businesses and administrative centres were completely closed. During this period, children on the spectrum received only online therapy support and worked with their parents at home. With this in mind, we believe that the results objectively show the real difference in emotional and behavioral challenges, regardless of the number of participants, as, when the first study was conducted, there was a complete lack of information about the forthcoming pandemic and the events, which would take place in the following two years.

Additionally, home schooling during the first lockdown was interesting to observe in terms of its impact on children’s behavior and emotional regulation, as neither the children, nor their parents were prepared for that experience. In this sense, we believe that observing children during this period would provide a more complete picture of the impact of staying at home and learning online on levels of emotional and behavioral problems.

### 3.2. Instruments

The participants filled out a battery of questionnaires in February 2020 and June 2020, including:

- Childhood Autism Spectrum Test (CAST) [34,35]. The questionnaire contains 39 yes/no questions for parents. CAST is a parent questionnaire that looks for signs of autism in the child, such as difficulties in social communication. The questionnaire is used to estimate the severity of symptoms exhibited by children on the spectrum. It has been demonstrated to be an effective screening tool for autism spectrum disorders (ASD) and social communication issues. In this study, we used the Bulgarian version of the questionnaire [36]. It was administered to both typically developing children and children with ASD in February 2020. The questionnaire can be administered to children with typical development to determine whether the scores correspond to no risk, present risk, or high probability of ASD symptoms. Further diagnostics are necessary if the results indicate a risk.
- Child Behavior Checklist (CBCL) [37,38,39], a questionnaire for parents, is a part of the Achenbach System of Empirically Based Assessment (ASEBA). ASEBA screens children, adolescents and adults for behavioral and emotional issues [40,41]. The CBCL was administered to both study groups in February 2020 and in June 2020, after the first lockdown, to children with ASD only.
- A survey designed to examine the influences of the COVID-19 pandemic on children’s lifestyle and development was administered to parents of children with ASD and parents of typically developing children in June 2020, after the first COVID-19 lockdown. The survey was compiled by the authors and included questions related to changes in the child’s behavior during the lockdown, educational issues, everyday life, mental well-being, as well as questions related to the parents’ own anxiety. The questions were open ones, i.e., parents were given the opportunity to share more information related to the children and their respective behavior during the lockdown. The answers of the parents were of particular interest to the research team because this was a unique situation for both children with developmental difficulties and typical development. The answers reflected the opportunity parents had to spend more time with their children at home, hence observing them better and interacting considerably more with them.

### 3.3. Participants

The research covers:

Group 1—children with ASD (N = 21; four girls and 17 boys; 48–62 months of age; medium age 54.62 months). All participants in the study had a previously established diagnosis of Childhood Autism—F84.0, according to ICD 10 and medical documentation corresponding to the state legislation of a child with Childhood Autism and special educational needs.

Group 2—typically developing children (N = 21; four girls and 17 boys; 48–60 months of age; medium age 53.81).

## 4. Results

### 4.1. Administration of Childhood Autism Spectrum Test and CBCL Questionnaire in Both Children with ASD and Typically Developing Children

The results of the initial administration of CAST for both groups were as follows: Children with ASD: mean/20.29/; SD/3.39/; median/20.00/. TD (typically developing) children: mean/5.43/; SD/1.94/; Median/5.00/, with statistically significant difference between the groups—Kruskal-Wallis test: *p* < 0.001.

The application of this questionnaire showed that all children in the ASD group have marked symptoms compared to the group of typical children.

The results of the initial administration of the CBCL questionnaire for both ASD and TD groups of children were as follows:

Internalizing Problems:

ASD children: mean/21.24/; SD/8.36/; TD children: mean/8.81/; SD/5.20/; Kruskal-Wallis test: *p* < 0.001.

Externalizing Problems:

ASD children: mean/20.24/; SD/9.39/; TD children: mean/10.00/; SD/4.44/; Kruskal-Wallis test: *p* < 0.001.

Total Problems:

ASD children: mean/61.62/; SD/25.14/; TD children: mean/26.48/; SD/13.16/; Kruskal-Wallis test: *p* < 0.001.

For all Empirically based scales in the subgroup of Internalizing problems—emotionally reactive, anxious/depressed, withdrawn, except somatic complaints, ASD children showed significantly higher scores compared to the TD children.

The differences for the scale sleep problems were not significant, although children with ASD displayed higher results than TD children.

For both attention problems and aggressive behavior in the subgroup of externalizing problems in the empirically based scales, ASD children displayed significantly higher results than TD children with a greater difference for the attention problems. The same was valid for the other problems scale.

In the subgroup of DSM-oriented scales: Depressive Problems, Anxiety Problems, Autism Spectrum Problems, Attention Deficit/Hyperactivity Problems, Oppositional Defiant Problems the ASD children showed significantly higher scores than TD children.

### 4.2. Comparison of the Results of Children with ASD of the CBCL Questionnaire before and after the First COVID-19 Lockdown

Results from scales belonging to the internalizing problems questionnaire subgroup—Table 1.

For all scales in this questionnaire subgroup, the values of internalizing emotional and behavioral problems after the first lockdown period were lower than those before the lockdown, with no statistically significant difference observed. Similar results had with the sleep problems scale—3.95 means before the lockdown and 3.81 means after the lockdown with no statistically significant difference.

The results from the externalizing problems scales are included in Table 2 and the results from the other problems scale are included in Table 3. We observed significantly lower results in the attention problems scale in the second application of the questionnaire.

Results from DSM-oriented scales—depressive problems, anxiety problems, autism spectrum problems, attention deficit/hyperactivity problems, and oppositional defiant problems—Table 4.

The results of all the scales in the second application of the test were lower than those before the lockdown, but only the results of the attention deficit/hyperactivity problems scale were statistically significantly lower.

Results from internalizing, externalizing and total problems—Table 5, where we did not find statistically significant differences.

The internalizing problems questionnaire subgroup consists of inward-directed behaviors. They are often underestimated and hardly noticed by teachers, compared to the externalizing ones, which usually create more problems, especially when interacting in a group [41,42]. Internalized behaviors are more often observed by parents and nonetheless underestimated as well [43]. A question of particular interest for the researchers regarding the ASD group was related to the internalizing problems, and in particular the relationship between them and the online education, as well as the forced stay at home, especially in the withdrawn scale, which comes closest to ASD symptomatic. According to the parents in the ASD group, internalizing behavioral problems, such as emotional reactivity, depression, somatic complaints and withdrawal, did not increase during the lockdown period. When drawing conclusions, we have to take into consideration parents’ own anxiety levels in these conditions, such as threat of illness for example [10]. Another consideration relates to that parents were deprived of the much-needed therapies and activities, related to their state in that period [44,45].

In the externalizing problems questionnaire subgroup, which do the adults usually more easily note, children with ASD did not display increase, contrary to our expectations. We presumed that the monotony and lack of exciting activities and therapy will lead to lack of concentration and energy excess. In our study, we observed quite the opposite—the rate of the behaviors, related to attention and hyperactivity decreased, without it resulting in a significant decrease in the externalizing behaviors scale. This effect was also present in the group of the empirically based attention problems scales, as well as the DSM scale attention deficit/hyperactivity problems. Practically, it means that parents reported improved behavior, lowered physical activity and more opportunities for keeping the children engaged in activities [46]. Perhaps, the strict and undisturbed routine, the interaction with parents, the time and patience that parents devoted to the children, as well as the lack of social pressure in a group, lack of stress meeting new people, despite the new and unknown conditions, played a positive role on both emotional and behavioral aspects of the children with ASD. Finally, yet importantly, the satisfaction and parents’ attention had their influence on the motivation, improved concentration, and engagement in home-based activities.

### 4.3. Results of a Parent Survey for the Consequences of the First COVID-19 Lockdown in June 2020

For the purpose of the present work, only some of the results are presented here. The included questions addressed emotional and behavioral problems. Questions related to educational issues, everyday life, and self-care were not included.

- Question 1: “Do you observe a decline in the social skills of the child as a result of the isolation during the COVID-19 lockdown, ability to communicate with adults and peers, or be part of a group and communicate successfully with other children?”

Parents of ASD children: Seven (7) parents answered “Definitely Yes” and seven (7) parents answered, “To a certain extent”. The other parents think that there was no decline or they are not sure.

**Figure 1:**
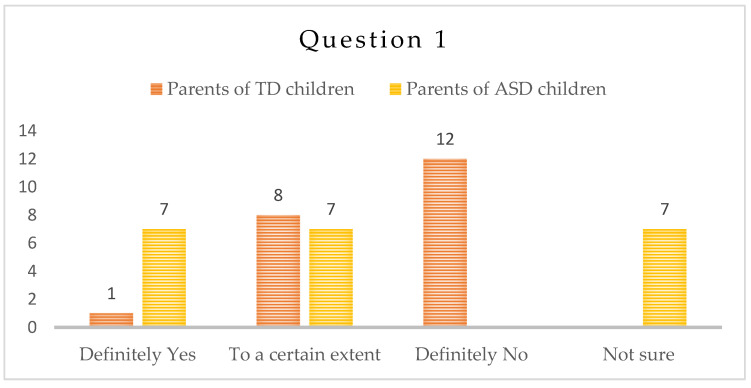
Number of answers of Question 1.

Parents of TD children: According to one (1) parent, the child displays lower level of social skills during the pandemic, and other eight (8) parents answered “To a certain extent”. The rest of the parents did not report change—Figure 1.

Social skills are a key characteristic of children with ASD. We expected a decline in these skills as a consequence of the isolation period, but parents only observed such a decline to a certain degree. Only a third of the parents confirmed a decline in social skills.

We expected the answers to the open questions to point towards social skills deterioration for both groups of children due to the lack of social communication in a lockdown period, especially the one with peers [47,48]. According to the participants in the study, the social skills of their children with typical development were not severely affected. Parents of children with ASD reported certain impact, but still not as significant as we expected. Perhaps, in children with ASD, this reporting referred to the loss of already acquired behaviors, a result of the intensive therapeutic interventions, work in groups and meetings with various people every day, beforehand.

In addition, there are several factors that might have affected the answers of the parents from the ASD group. Children with ASD already displayed significant difficulties in the acquisition and use of social skills, and therefore a change was more difficult to note. In addition, an acquired skill or behavior in therapeutic settings, if not generalized, would not be demonstrated by the child in a real-life situation. Last but not least, we have to bear in mind that parents’ opportunities for observation of their children’s interactions with peers after the lockdown were limited.

- Question 2: “Do you think that the language skills of the child declined as a result of the isolation during the COVID-19 lockdown—the ability to use words, apply grammatical rules, etc.?”

About half of the parents of children with ASD/n = 12/did not consider that their children’s language skills worsened as a result of the lockdown and one (1) of them is not sure. The rest of the parents are equally divided between the answers “Yes” and “To a certain extent”.

**Figure 2:**
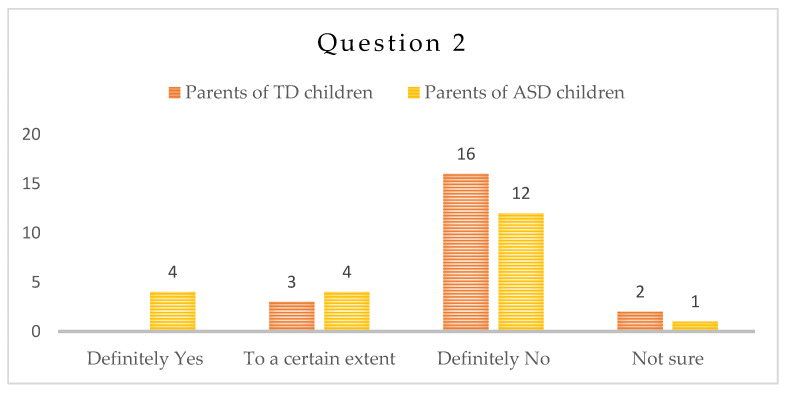
Number of answers to Question 2.

Most parents of typically developing children/n = 16/report that the language abilities of their children did not deteriorate during the pandemics. Three parents/3/noted some deterioration and two/2/parents stated they are not sure they did—Figure 2.

Speech and language ability is also a key skill for the children with ASD. They are of utmost importance for the development of communication and social skills, increasing the opportunities for the children to express themselves and understand the others leads to less behavioral problems and easier adaptation. Communication with peers plays a major role in this context. We expected the children with ASD to display deteriorated language skills during the lockdown period, which, according to the answers of the parents, did not prove right. A possible reason for this could be that they were given the opportunity to stay at home together and interact in new ways for the first time. We believe that parents experienced a shift towards the new role of a co-therapist, because in this situation they had to—under the guidance and directions, given by the therapist—revert to and invent new ways of work with the child in home settings, e.g., embed therapeutic activities within home routines and thus practice existing and acquire new language skills. The answers to this and other questions in the frame of this study point to the more relaxed atmosphere, the opportunity to interact with the child at home, the lack of strict schedule of therapies as possible reasons for the perceived benefits for the children with ASD and shift the focus from the lack of fully structured yet useful activities.

- Question 3: “Do you observe, as a result of the isolation during the COVID-19 period, an increase in the anxiety levels of the child?”

**Figure 3:**
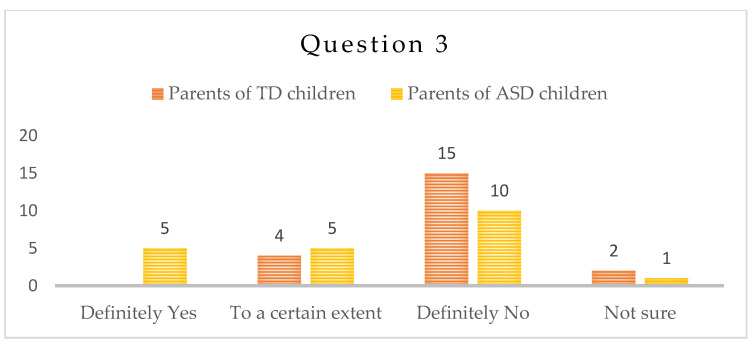
Number of answers to Question 3.

Ten/10/of the parents of children with ASD gave a negative answer to this question, and one was not sure. The rest were equally divided between “Yes” and “To a certain extent”. Parents of typically developing children had answered “No”—fifteen (15), “I am not sure”—two (2), and “To a certain extent”—four (4)—Figure 3.

Anxiety plays a major role in the functioning of the children with ASD [49,50]. We relate anxiety to the obsessive attachment to routine and stereotypical movements in children with ASD and consider its reduction to be an important aspect of the therapy. As expected, according to the results, anxiety in children with ASD showed lower values, probably due to the less intensive daily routine, seldom change of places, therapists, hurry and commute in heavy traffic. The restriction in activities and lack of new ones largely eliminated the anxiety trigger. Parents of children with special needs would enroll them in many and diverse intense therapies, that are, in most cases, exhausting for the children. Perhaps the forced stay at home had taken away part of this pressure, and at the same time, interaction with parents had played its positive role. As a result, children with ASD did not display an increase in anxiety levels during the pandemics.

- Question 4: “Did the child become more irritable during the isolation period during the COVID-19 pandemic?”

**Figure 4:**
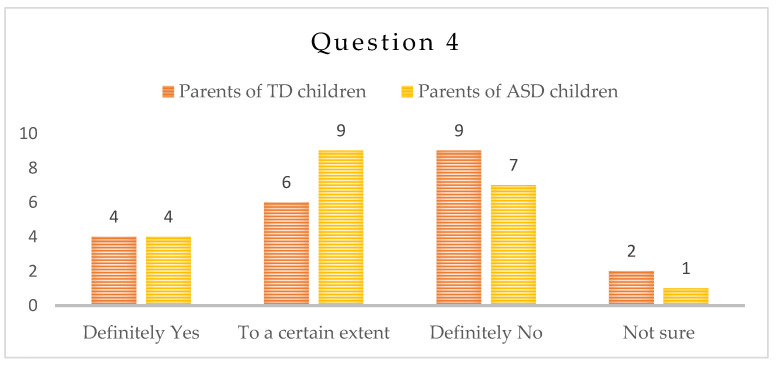
Number of answers to Question 4.

Nine (9) parents of children with ASD have confirmed that their children have become more irritable to a certain extent, and four (4) parents definitely noted increased irritability; seven (7) parents did not find any difference and one (1) was not sure. In the group of typically developing children, four (4) parents find their children more irritable during the pandemics, six (6) parents reported increased irritability to a certain extent, nine (9) have answered negatively and two (2) were unsure—Figure 4.

Irritability, expressed in behavioral aspect, is easier to be observed by parents. One of the most common complaints on behalf of the parents in clinical practice are the emotional crises of their children. In both groups of parents, the answer to this question was predominantly “To a certain extent” and “No”, which might be partially due to the increased time at home with the children.

In addition, for the children in the ASD group, we may attribute the result to less exposure also to external triggers for irritability and predictable routine, due to the lockdown situation. Finally, less irritability and anxiety lead to a more efficient learning process in the aspect of social communication and speech and language ability, which in turn is very satisfactory for both children and parents.

- Question 5: “Did your own anxiety levels increase during the isolation period?”

**Figure 5:**
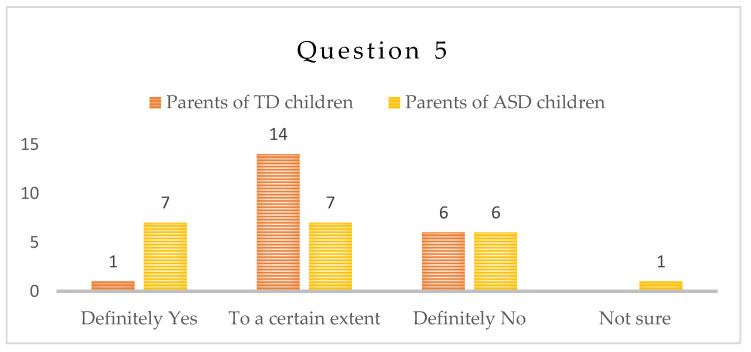
Number of answers to Question 5.

This was a very important question for the parents and it tackled their own anxiety levels. In the group of children with ASD, seven (7) parents report increased anxiety levels, seven (7) thought that it has increased to a certain extent, six (6) answered negatively and one (1) was unsure. In the group of TD children, one (1) parent reported increased levels of anxiety, fourteen (14) thought that it had increased to a certain extent, and six parents (6) did not observe any increase in their own anxiety levels—Figure 5.

The mental well-being of the parents is a major factor in the development of their children [51,52]. Increased parental anxiety has a negative effect on the overall state of the children and affects the outcomes of therapeutic interventions. The long stay at home during lockdown triggers worries about the future, fear of possible health issues and unclear consequences for the children, which may have negative effect on the care of children with developmental difficulties. This research points to a general lack of support for parents concerning the online work with their children. This support needs to be carefully designed, with all possible threats of uncertainties of the future in mind.

Parents’ Answers to Open Questions

Instruction: “Please share other consequences of isolation at home during COVID-19 pandemic”.

Typically developing children

In general, parents of children with typical development shared only negative consequences of the isolation period: lack of opportunities to go out, less physical activity, overuse of electronic devices, and lack of social interaction and contact. When responding to the open-answer questions, no parent pointed to any positive effect on the child during the lockdown period.

Children with ASD

Parents of children with ASD gave both positive and negative comments. Their answers are presented in Table 6.

## 5. Discussion

The open answers of the parents of children with ASD to the questionnaire showed that the effects of isolation and time at home were more positive than they were for typically developing children. Some of the parents reported that they had had time to practice various skills with their children that required patience and multiple repetitions. A major negative consequence of the lockdown for children with ASD was an increase in the time spent in front of electronic devices, which is congruent with previous studies [53]. For families of children with ASD, lockdown situation provided time that they spent together, that contributed to the acquisition of new skills. Some studies have demonstrated that interactions between children with ASD and their parents are more likely to result in beneficial outcomes, such as a development in the child’s communication skills, than interactions between children and their instructors. Hence, more child-parent interactions may have averted a decline in communication skills during the lockdown [54].

Behavioral changes in individuals with ASD are expected to occur as a result of the pandemic, and these changes are likely to have long-term effects on functioning [55]. However, some authors did not observe higher levels of problematic behaviors in ASD children due to the pandemic [56]. These differences could be due to various factors related to the environment and the way parents responded to the pandemic conditions. It is possible that in the cases where the family managed to turn the time at home into a positive factor—more time for communication, application of interventions at home, time for fun and mutual activities with the child, slow introduction of the educational material and time for training in daily activities, the children showed fewer behavioral and emotional difficulties. This underscores the enormous importance of the parents involvement in the interventions for children with ASD and possibly the importance of the still underestimated effect of distance communication with parents and the resources of telepractice.

Repetitions, lack of pressure, related to time, traffic, daily schedule, provided the time needed for parents and children to acquire new skills, learn and play. The lessons to be learned from the lockdown situation for the children with ASD relate to more opportunities for parents and children to interact and communicate, learn new skills for self- care, build patience for more repetitions and open more learning opportunities. In this aspect, one of the tasks in the list of the professionals is to take care of the mental well-being of the parents, so they are a better support for their children.

It is possible that during the lockdown the children with ASD in our group had more opportunities to concentrate on learning, to decrease the stress of everyday social challenges they face, and to use technologies. One important fact to point out is that we had not yet fully resumed normal operations after the first lockdown in June 2020, which is why we presume that parents did not have the opportunity to observe their children’s functioning in different situations—at the kindergarten, nursery, children’s parties, when playing, etc. We do not have the opportunity to observe the long-term effects, and the results here are only indirect, immediately after the onset of the pandemic. The long-term effects would probably be more negative than the immediate ones. However, if we consider the results of this study and analyse them, we will appreciate and understand better the major role of the parent in the child’s life and especially those with ASD and manage the long-term prospective better. Instead of multiple chaotic therapeutic interventions, we should train parents in skills that will allow them to work and go through the learning process with their children more effectively, something that should be done in all settings, with materials at hand and in a playful context. Children and parents should enjoy more quality time together—sharing activities and learning together, that will have beneficial effect for both in the long-term prospective.

## 6. Limitations of the Study

The small sample group of children has limits when it comes to drawing stable conclusions about the effects of isolation during the lockdown on the emotional and behavioral aspects of the functioning of children with ASD. Nevertheless, the children in our study are placed in a unique situation, having been observed immediately before and after the first lockdown period, which, per se, was a challenging and unusual situation that no one expected or was prepared for.

Another limitation is the use of only questions to the parents who evaluate their children’s behavior which suggests that results could be influenced by their subjective opinion. In the current situation, parents’ opinion may also be influenced by their own anxiety and personal perception of the pandemic situation.

## 7. Conclusions

The unprecedented opportunity for comparison of the assessment data of problematic behavior of children with ASD before and after the first lockdown gave us the chance to compare, with certainty, the differences in the situation, measured with a single instrument. The results of all scales lead to the conclusion that after three months at home, children with ASD did not demonstrate more problematic behavior. Although in Bulgaria the professionals did not have much experience in online services, the fact that parents stayed at home and spent more time with their children probably influenced both groups rather positively. Many parents of children with ASD try to provide the best services for their children, which probably relates to overload and lack of time for even basic communication.

To conclude, we note that the following approaches should be given more attention in the care of ASD children:

First, the relationship between the parents and the child should be established, strengthened, and supported. Additional support is needed from therapists in this task. Telemedicine methods are considered to be an acceptable and useful approach to enhancing communication between parents and therapists [57].

Secondly, a revision of children’s programs and the provision of more time for free communication are needed. Parents need support from professionals in the form of advice and programs to enhance communication at home.

Third, we recommend more technologies for work at home or at school, if they are not overused and their introduction in the process is controlled and related to the acquisition and development of knowledge and skills with the participation of the parents, promoting the communication between children and relatives.

## Figures and Tables

**Table 1:** Comparison of the results from Internalizing problems of children with ASD before and after the first lockdown.

CBCL	ASD Children before the 1st Lockdown	ASD Children after the 1st Lockdown	Kruskal-Wallis Test
Emotionally Reactive			*p* > 0.05
Mean	6.43	6.28	
St. dev.	3.20	3.24	
Anxious/Depressed			*p* > 0.05
Mean	5.86	5.38	
St. dev.	2.76	2.84	
Somatic Complaints			*p* > 0.05
Mean	3.14	2.90	
St. dev.	2.41	2.45	
Withdrawn			*p* > 0.05
Mean	5.81	5.57	
St. dev.	2.77	2.68	

**Table 2:** Comparison of the results from the externalizing problems in children with ASD before and after the first lockdown period.

CBCL	ASD Children before the 1st Lockdown	ASD Children after the 1st Lockdown	Kruskal-Wallis Test
Attention Problems			*p* < 0.05
Mean	6.14	4.71	
St. dev.	2.10	1.55	
Aggressive Behavior			*p* > 0.05
Mean	14.08	13.14	
St. dev.	7.78	7.73	

**Table 3:** Comparison of the results from the other problems scale in children with ASD before and after the first lockdown period.

CBCL	ASD Children before the 1st Lockdown	ASD Children after the 1st Lockdown	Kruskal-Wallis Test
Other Problems			*p* > 0.05
Mean	20.14	19.28	
St. dev.	8.89	8.80	

**Table 4:** Comparison of the results from the DSM-oriented scales—children with ASD before and after the first lockdown period.

CBCL	ASD Children before the 1st Lockdown	ASD Children after the 1st Lockdown	Kruskal-Wallis Test
Depressive Problems			*p* > 0.05
Mean	2.62	2.19	
St. dev.	2.15	2.13	
Anxiety Problems			*p* > 0.05
Mean	4.90	4.62	
St. dev.	2.37	2.32	
Autism Spectrum Problems			*p* > 0.05
Mean	11.48	11.00	
St. dev.	4.94	4.76	
Attention Deficit/Hyperactivity Problems			*p* < 0.05
Mean	10.52	8.57	
St. dev.	3.51	3.06	
Oppositional Defiant Problems			*p* > 0.05
Mean	5.67	5.09	
St. dev.	2.96	2.94	

**Table 5:** Comparison of the results for the Internalizing, Externalizing and Total problems.

CBCL	ASD Children before the 1st Lockdown	ASD Children after the 1st Lockdown	Kruskal-Wallis Test
Internalizing			*p* > 0.05
Mean	21.24	20.14	
St. dev.	8.36	8.71	
Externalizing			*p* > 0.05
Mean	20.24	17.86	
St. dev.	9.39	8.86	
Total Problems			*p* > 0.05
Mean	61.62	57.28	
St. dev.	25.14	8.86	

**Table 6:** Answers to open questions of the parents of ASD children.

Positive Comments	Negative Comments
“The child did well with the tasks”.	“We are together all the time; I consider it negative”.
“I would not say there were negative consequences; for us, the result was positive—it gave us the opportunity to work together, to take the Pampers off, and the child learnt to eat by himself. We only missed the sessions with the speech pathologist”.	“He always needs an electronic device to stay still”.
“My child was happy because she was not attending kindergarten. We progressed a lot, physically and emotionally; she worked and developed. It was a wonderful period for us!”	“He became naughtier and gained weight”.
“There were no consequences for us”.	“We observed increased anxiety levels and lack of physical activity”.
“She learned how to report her physiological needs (she wears Pampers). She repeats two to three words now in a sequence and imitates”.	“Overuse of tablet”.
“Working from home gave me more opportunities to share time with my child”.	“Anxiety, nervousness, and lack of physical activity”.
“My child has progressed a lot. I consider it a result of closer contact with the family, although he is not willing to sit and work. We talk all the time now and we try in a playful context to learn”.	

## Data Availability

Data supporting reported results can be obtained from the authors.
